# The Genetic Landscape of Fiber Flax

**DOI:** 10.3389/fpls.2021.764612

**Published:** 2021-12-07

**Authors:** Maria Duk, Alexander Kanapin, Tatyana Rozhmina, Mikhail Bankin, Svetlana Surkova, Anastasia Samsonova, Maria Samsonova

**Affiliations:** ^1^Mathematical Biology and Bioinformatics Laboratory, Peter the Great St. Petersburg Polytechnic University, Saint Petersburg, Russia; ^2^Centre for Computational Biology, Peter the Great St. Petersburg Polytechnic University, Saint Petersburg, Russia; ^3^Laboratory of Breeding Technologies, Federal Research Center for Bast Fiber Crops, Torzhok, Russia; ^4^Institute of Translational Biomedicine, Saint Petersburg State University, Saint Petersburg, Russia

**Keywords:** population genetics, flax, genetic diversity, gene flow, breeding history, crop improvement, heritage landraces

## Abstract

Genetic diversity in a breeding program is essential to overcome modern-day environmental challenges faced by humanity and produce robust, resilient crop cultivars with improved agronomic characteristics, as well as to trace crop domestication history. Flax (*Linum usitatissimum*), one of the first crops domesticated by mankind, has been traditionally cultivated for fiber as well as for medicinal purposes and as a nutritional product. The origins of fiber flax are hidden in the mists of time and can be hypothetically traced back to either the Indo-Afghan region or Fertile Crescent. To shed new light on fiber flax genetic diversity and breeding history, in this study, we presented a comprehensive analysis of the core collection of flax (306 accessions) of different morphotypes and geographic origins maintained by the Russian Federal Research Center for Bast Fiber Crops. We observed significant population differentiation between oilseed and fiber morphotypes, as well as mapped genomic regions affected by recent breeding efforts. We also sought to unravel the origins of kryazhs, Russian heritage landraces, and their genetic relatedness to modern fiber flax cultivars. For the first time, our results provide strong genetic evidence in favor of the hypothesis on kryazh’s mixed origin from both the Indo-Afghan diversity center and Fertile Crescent. Finally, we showed predominant contribution from Russian landraces and kryazhs into the ancestry of modern fiber flax varieties. Taken together, these findings may have practical implications on the development of new improved flax varieties with desirable traits that give farmers greater choice in crop management and meet the aspirations of breeders.

## Introduction

**FIGURE 1:**
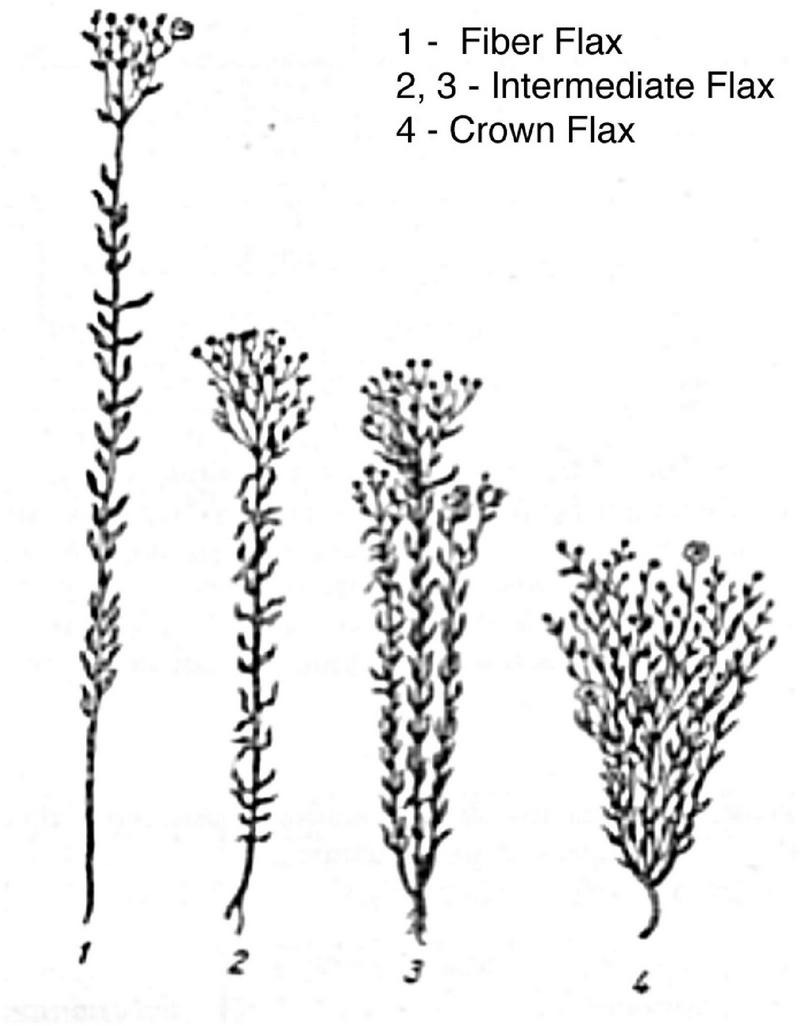
Flax morphotypes. Sketch of flax morphotypes, where intermediate and crown flax cartoons represent oilseed variety.

Flax is one of the oldest domesticated crops grown worldwide and in various climates. As centuries passed, a diverse range of breeding routes eventually gave rise to two very different phenotypes, namely (a) to a bushy, relatively small, short plant with high seed yield ideal for oil extraction (oilseed or linseed flax) and (b) to a fiber flax, long pliable unbranched stem crop with long cellulose-rich fibers best suited for linen production (Figure 1). Flax seeds are an excellent source of oil with high content of unsaturated fatty acids, lignins, easily digestible proteins, dietary fiber, vitamins, and mineral elements. Linseed oil is an important industrial commodity used for various purposes, including as a major constituent in paints, resins, printing inks, varnishes, and, finally, linoleum. Historically, fiber flax varieties have been used as a base material to produce textiles. However, recent advances in material science have led to renewed interest in fiber flax, as its fibers are now used in a wide range of environmentally friendly industrial applications, e.g., composites, geotextiles, insulation, and specialty papers.

Flax domestication history and its spread remain elusive; it is commonly believed, however, that oilseed flax was domesticated in Fertile Crescent in Neolithic times. Archeological research confirms that flax was known as far back as 8,000–9,000 years to inhabitants of Tell Ramad in Syria, where enlarged seeds, presumably, a result of primitive cultivation efforts, were found (van Zeist and Bakker-Heeres, 1975). Other dominant regions of flax diversity, as identified by Vavilov (1926, 1951), include the Indian subcontinent, Abyssinia, and the Mediterranean, where flax was bred from the same wild parent plant *Linum bienne*, most probably in geographic isolation to create the domesticated form of *L. usitatissimum.* Therefore, these regions may be associated with multiple domestication events of the crop. The first flax varieties to appear in Southern Europe (Danube valley) were winter oil crops (Allaby et al., 2005). Evidence from Africa and Europe suggests that by 4,000 BC, bast fiber textiles have spread to the Nile Valley and as far as modern-day Britain. Interestingly, in Eastern Europe, spring-planted fiber varieties emerged either as a result of independent domestication event in Central Asia (Indo-Afghan region) and subsequent diffusion (Supplementary Figure 1) or from European varieties found in the south (Helback, 1959; Sinskaya, 1959; Zhukovsky, 1971; Diederichsen and Hammer, 1995).

At the beginning of the 20th century, Russia became the main supplier of fiber flax of the highest quality to the European market, producing the crop in the northern regions of the empire. The cultivation efforts carried out by local peasantry eventually resulted in the development of Russian heritage landraces, also known as “kryazh” (plural: kryazhs). Kryazh forms emerged under both natural and artificial selection carried out by Russian peasantry for centuries, aimed at the development of tall fiber flax varieties with the longest possible elementary fibers. Kryazhs are a product of mass selection method known as “cropping.” The upper part of the tallest plants was chopped off during threshing process, and the seeds were used for sowing in the next year. The resulting fiber flax varieties are highly adapted to local agroclimatic conditions, probably due to the co-selection of adaptive genomic regions containing epigenetic information essential for the overall stability îf genotype and phenotype. Until the thirties of the last century, more than 100 kryazh forms were cultivated on the territory of Russia. Naturally, kryazh flax varieties were regarded as high commodity crops and thus were actively traded and used in breeding programs in many countries. It is widely accepted that all modern fiber flax varieties have Eastern European origin (Helback, 1959); however, the question of their relation to kryazhs remains open.

By 1970, 12 flax diversity regions were determined (Zhukovsky, 1968). Unsurprisingly, flax adaptation to different agroecological environments and cultural practices in these areas resulted in very distinct phenotypes. Large-seeded flax is common in the Mediterranean area, while crown flax varieties occur in Central Asia and the Indian subcontinent. Intermediate flax, the most common oilseed variety, has branched stem, medium height, plenty of flowers, and seeds (Figure 1). Fiber flax varieties are tall unbranched crops that are grown at very high density to maximize fiber production. It is commonly assumed that agroecological conditions of Eastern Europe, i.e., humid temperate climate, long daylight hours, and well-drained medium-heavy soils, favor the development of fiber flax (Marchenkov et al., 2003). Moreover, it is likely that agriculture practices made a significant contribution to the development of fiber flax varieties, in particular, as fiber crops are planted with a much higher density than oil plants. A remarkable phenotypic plasticity and its amenability to modern molecular genetic techniques make flax an attractive model to study crop adaptation mechanisms to both local agricultural practices and diversifying selection.

Rapid growth of the cotton-based textile industry in the 20th century decelerated fiber flax production, thus making oilseed flax more economically important than fiber flax. As a result, motivated by the high nutritional value of oilseed flax, the industry sector shifted its priorities to seed and oil production. More recently, though, the burgeoning interest in natural fibers for a variety of industrial uses fueled the development of new fiber flax varieties (Goudenhooft et al., 2017).

Evidently, a detailed characterization of the plant genetic makeup and crop diversity are key components for the success of the breeding programs. The collections across the world contain over 40,000 accessions of cultivated flax (FAO, 2010). However, estimates show that only 10,000–15,000 of them are unique (Diederichsen, 2007). Over the years, the diversity in flax germplasm has been delineated using different technologies and markers, including RADP, SSR, retrotransposon-like, and SNP (Fu, 2005; Smýkal et al., 2011; Soto-Cerda et al., 2012, 2013; Rozhmina et al., 2018). These studies primarily encompassed accessions from the Canadian national seed bank at Saskatoon (2,813 accessions of cultivated flax from 69 countries). The main findings accumulated in the recent years are summarized as follows: (a) cultivated flax varieties exhibit considerable genetic diversity congruent with cataloged morphotypes, (b) genetic differentiation between fiber and oilseed flax groups is not well pronounced, (c) a rapid decay of the linkage disequilibrium (LD) is exhibited, and (d) geographic patterns of diversity are in agreement with dominant regions reported by Vavilov, as accessions from the Indian subcontinent and Africa form two genetically distinct groups.

This study challenges some of the aforementioned results, expands current knowledge on the genetic diversity of flax, as well as provides novel insights into the breeding history of the crop by extensive characterization of a representative subset of the collection maintained by the Federal Research Center for Bast Fiber Crops. A wealth of genetic information on cultivars, breeding lines, and heritage landraces generated in this study is undoubtedly of critical importance for the advance of crop improvement programs as well as for the success of flax breeding schemes.

## Materials and Methods

### Plant Material Collection and DNA Sequencing

A total of 306 flax accessions of different morphotypes and geographic origins were selected from the collection of the Federal Research Center for Bast Fiber Crops (Supplementary Table 1). The dataset contained 182 fiber flax accessions, 120 linseed flax accessions, and 4 unknown morphotype accessions. The oilseed group was further classified into the following subgroups: 99 intermediate accessions, 5 large-seeded accessions, and 16 crown accessions. The selection status of accessions in the dataset was as follows: landraces, elite cultivars, and breeding lines. Notably, 30 fiber accessions and 1 intermediate accession belonged to “kryazhs,” and the heritage landraces were developed by Russian peasantry (Supplementary Tables 1, 2). The geographic origin/release of the accessions encompassed 30 countries and all continents. All plants were grown in the experimental field of the Federal Research Center for Bast Fiber Crops in Torzhok, Russia. DNA was extracted from collected leaves using DNeasy Plant Mini Kit (Qiagen, Santa Clara, CA, United States). DNA sequencing was performed at the Beijing Genomics Institute (BGI) using Illumina protocol generating paired-end reads of 150 bp in length. Processed reads were aligned to NCBI flax reference genome assembly ASM22429v2 with bwa-mem using default parameters (see Supplementary Table 3 for details on sequencing and alignment) (Li and Durbin, 2009). Variant calling was run using NGSEP (Tello et al., 2019) version 4.0. Flax genome annotation (You and Cloutier, 2020) was kindly provided by the Cloutier group (Ottawa Research and Development Centre).

### Population Structure Analyses

Patterns of population differentiation were examined using the Uniform Manifold Approximation and Projection (UMAP) algorithm (McInnes et al., 2018) as implemented in R package umap. Following dimensionality reduction, the data were scaled and clustered (k-means) to reveal groups. The clustering quality was evaluated using Silhouette plots (Supplementary Figure 2B). The genetic structure in the dataset was estimated using the ADMIXTURE software (Alexander et al., 2009). The analyses were performed for *K* values ranging from 2 to 6, and *K* = 4 was selected according to the minimal value of the cross-validation error. To analyze relationships among genotypes and construct maximum likelihood phylogenetic trees, we used SNPhylo (Lee et al., 2014) with MAF filtration threshold of 0.01. The trees were drawn using ggtree R package (Yu, 2020). The pairwise relatedness between accessions was estimated by calculating kinship coefficients in popkin R package (Ochoa and Storey, 2021). Patterson’s *D*-statistic (ABBA-BABA) on genome-wide SNP data was used to infer patterns of introgression (Green et al., 2010; Durand et al., 2011). *D*-statistic was calculated using AdmixTools (Patterson et al., 2012) with qpDstat program. To compute *D*-statistic, we used intermediate flax varieties from America as outgroup (population *Z*) and intermediate flax varieties for Eurasia as one of the populations tested for introgression (*X*). Positive *D*-statistics points on the excess of alleles shared between population *W* and admixing population *Z*.

### Linkage Disequilibrium Analysis

The LD in five different flax accessions groups, as well as in all accessions, was evaluated using squared Pearson’s correlation coefficient (*r*^2^). The PopLDdecay (Zhang et al., 2019) version 3.4.1 was used to calculate *r*^2^ using the following filtration parameters: MAF > 0.05, missingness = 0.75 for pairwise markers in a 500 kb window. The LD decay was calculated based on *r*^2^ and the distance for each pair of SNPs using an R script. The *r*^2^ estimates were transformed with nonlinear square root according to Hill and Weir model (Hill and Weir, 1988) to approximate the distribution. A value corresponding to a half of maximum *r*^2^ value of 0.62 was considered as evidence of linkage. The intersection of the approximation curve fit to the *r*^2^ with this baseline was considered as the estimate of the extent of LD in the chromosome.

### Nucleotide Diversity and Population Differentiation Metrics

The nucleotide diversity (π), population differentiation statistics Fst (Hudson et al., 1992; Holsinger and Weir, 2009), and Tajima D (Tajima, 1989) were estimated from polymorphic sites in 10 kb nonoverlapping windows for each chromosome using VCFtools (Danecek et al., 2011). Group comparisons were performed either by contrasting median π-diversity values calculated for each chromosome or by estimating the reduction of diversity (ROD) which is the ratio of diversity between accession groups under comparison. ROD values were calculated across genome for each 10 kb nonoverlapping windows using the following equation:


$$R\,O\,D=1-\pi_{g\,r\,o\,u\,p\,2}/\pi_{g\,r\,o\,u\,p\,1},$$

where π is the average number of nucleotide differences between any two genomes within the group.

To identify possible regions under selection across various morphotypes, we compared breeding lines and cultivars separately with either landraces or kryazhs. In each comparison, we calculated both ROD and Fst statistics and selected regions with substantially low diversity values using both. Regions with significantly high population differences between two groups (highest values of Fst, top 5% of the whole genome, and top 2.5% values of RÎD) were considered as possible candidate regions under selection.

### Identity by Descent

PLINK package was used to quantify the identity by descent (IBD) sharing probability (Purcell et al., 2007). A pair of accessions was considered to be related if the value of pi_hat parameter was higher than 0.5. The relatedness graphs were drawn using Gephi software (Bastian et al., 2009). Due to a large number of connected accessions, the IBD values were further filtered using the pi_hat threshold of 0.7547 that was established from pairwise IBD relation between RuCer1581 kryazh and elite cultivar Svetoch (RuSve2843), a direct descendant of RuCer1581.

## Results

### Germplasm Sequencing and Genome-Wide Variation

Resequencing of 306 flax accessions resulted in 1,143.850625 Gb of raw data comprising 7.626 billion reads with an average of 9.3 × genome coverage or, alternatively, 3.7 Gbp yield per sample (Supplementary Table 3). Alignment of clean reads to flax reference genome assembly GCA_000224295.2_ASM22429v2 resulted in 11.7% horizontal genome coverage. Variant calling and filtering with MAF = 0.01 identified 3,416,829 biallelic SNPs. Average SNP density per 1 Mb interval was similar among chromosomes (Supplementary Table 4). The majority of SNPs were present in intergenic regions and only 9.5% of the variants mapped to coding regions. The ratio of non-synonymous to synonymous SNPs varied between accessions, and average ratio was 0.82 (Supplementary Table 5).

### Genetic Structure of Flax Population

**FIGURE 2:**
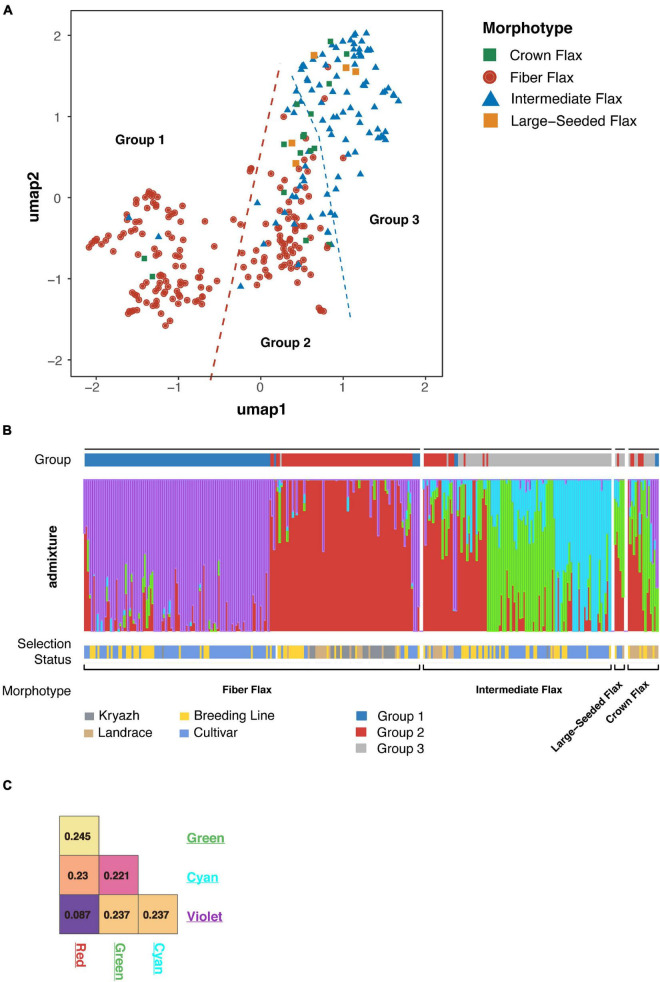
Population structure of flax accessions. **(A)** Visualization of flax accession clustering using Uniform Manifold Approximation and Projection (UMAP) algorithm. Fiber flax accessions are shown as red dots, intermediate flax accessions are blue triangles, crown flax and large-seeded accessions are presented as green and yellow squares, respectively. **(B)** The ADMIXTURE graph identified four subpopulations and supports subdivision of sequenced accessions into three groups. **(C)** Subpopulation differentiation calculated with Fst statistics. Color labels correspond with the populations revealed by admixture.

Flax has a diverse and complex history of domestication and breeding dating back to the hunter-gatherer era and traceable as far as Holocene Climate Optimum (*People, Plants, and Genes: The Story of Crops and Humanity*). We utilized a number of analyses to study the genetic structure in flax populations of different origins, including the UMAP algorithm and ADMIXTURE. Both methods revealed a remarkable degree of differentiation across the morphotypes (Figure 2 and Supplementary Figure 2). We subdivided accessions into three groups. The largest group encompassing ∼30% of the genotypes consisted of fiber breeding lines and cultivars, including two linseed landraces and one kryazh (Figure 2A and Supplementary Table 1). The second group included 76 fiber flax accessions, 11 intermediate flax cultivars and breeding lines, and 13 linseed landraces. Moreover, the group was comprised of nearly all kryazhs. Finally, the last group of genotypes was mainly made of oilseed flax (84 intermediate flax accessions), with a tiny exception of three fiber flax accessions. ADMIXTURE analysis showed four subpopulations that supported UMAP-driven partition of the collection into three groups (Figure 2B). The majority of accessions forming the first and second group belonged to subpopulations marked with violet and red, while the third group drew together genotypes attributed to the other two subpopulations (i.e., green and cyan). The allelic admixture was especially evident in the latter group, as a considerable number of accessions was a mixture of cyan and red, red and cyan, or red and green subpopulations. The weakest population structure as evidenced by the coefficient of population differentiation (Fst = 0.087) was observed between red and violet populations (Figure 2C), thus reflecting a common breeding history of fiber flax accessions from these groups. In sharp contrast, green and cyan subpopulations were distinct from the other two and were well differentiated from one another.

**FIGURE 3:**
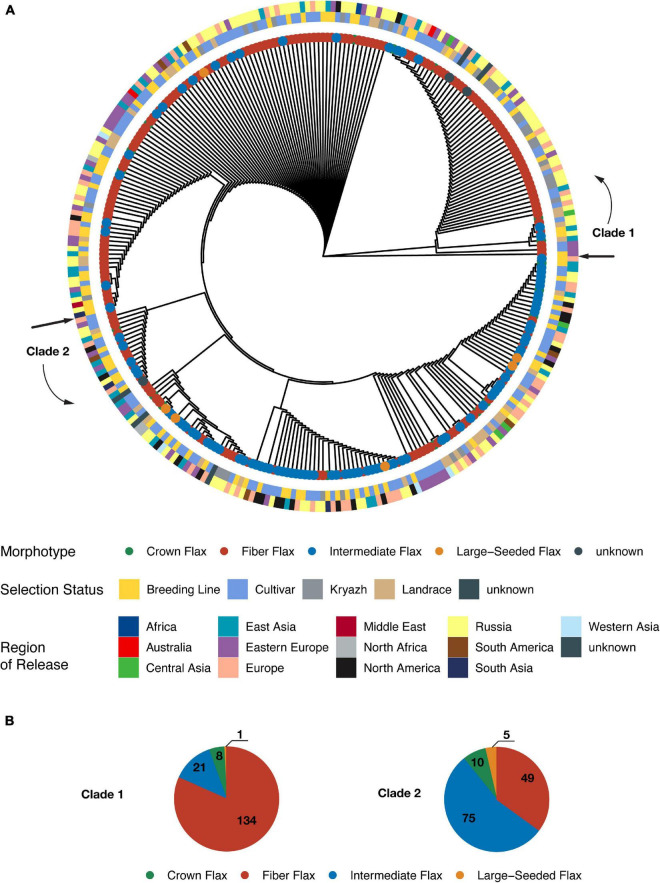
A maximal likelihood phylogenetic tree constructed from genome-wide SNPs. **(A)** The circular cladogram. The outermost ring depicts distribution of accessions according to country or region of release. The next two rings show grouping of accessions with respect to selection status and morphotype, respectively. **(B)** Pie charts showing composition of the clades. Each slice represents a particular morphotype.

A maximum likelihood phylogenetic tree presented two distinct clades (Figure 3A and Supplementary Figure 3), as very few intermediate and large-seeded accessions clustered with fiber flax accessions (30 samples out of the total of 165). While we observed opposite types of genotype placement, only a relatively small number of fiber accessions belonged to the oilseed clade (49 fiber genotypes, as opposed to 90 oilseed specimens, see Figure 3B). No obvious clustering of accessions with respect to their selection status or geographic origin was observed (adjusted Rand indices of −0.002 and 0.011, respectively) indicating a substantial movement of germplasm. Interestingly, landraces were interspersed between genotypes of both clades, while the vast majority of kryazhs were grouped within fiber flax clade. This observation was further supported by kinship analysis (Supplementary Figure 4), as (a) accessions associated with fiber flax varieties formed distinct cluster and (b) average kinship coefficient for fiber flax was 0.43, reaching a value of 0.46 for the kryazhs and landraces group. In contrast, average kinship coefficient computed for non-fiber morphotypes was 0.28.

### Linkage Disequilibrium Decay and Genetic Diversity

**FIGURE 4:**
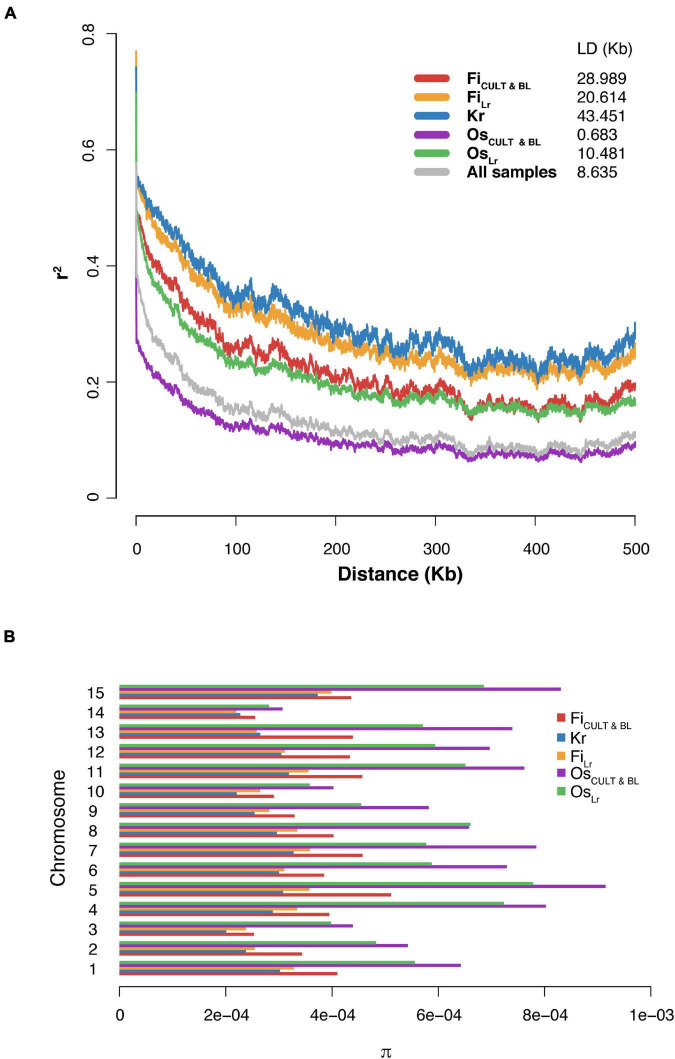
The LD decay and genetic diversity in groups of accessions with different selection status. **(A)** Graphs of the LD decay. **(B)** Variation of the median nucleotide diversity value between groups presented by chromosomes.

As expected, and in agreement with previous studies (Guo et al., 2019), the LD decays to half of maximum *r*^2^ value at a distance of 8.64 kb (as estimated for the whole dataset). Also, the LD decay rate for fiber genotypes was significantly slower than oilseed accessions (Figure 4A). A noticeable difference in the LD decay values was observed between accessions with different selection statuses. The observation was valid for both fiber and oilseed varieties. Importantly, the LD decay in flax accessions observed in this study was much faster than those detected for cultivated soybean (150 kb) (Zhou et al., 2015) and for cultivated rice (123 kb for *indica* and 167 kb for *japonica*, respectively) (Huang et al., 2010; Xu et al., 2011).

An evaluation of global nucleotide diversity (π) across different flax groups (Supplementary Tables 2, 6) revealed that the median value of nucleotide diversity in both oilseed cultivars and landraces was approximately two times as high as in fiber flax accessions (Figure 4B and Table 1), where kryazhs were least diverse. This trend was also demonstrated on the chromosomal level, and the largest difference between linseed and fiber groups was identified for chromosomes 4, 5, and 15 (Figure 4B and Supplementary Table 6). No significant difference in nucleotide diversity between morphotypes or subtypes pooled by geographic origin was detected. The negative Tajima *D* values calculated on all 15 chromosomes in all flax morphotypes suggested either a population size expansion (e.g., due to bottleneck) or a purifying selection in the course of crop diversification and improvement.

**TABLE 1:** Diversity levels among different flax groups.

Groups by		Diversity, π statistics
Morphotype	Fiber flax	0.0007401
	Crown flax	0.0009394
	Large-seeded flax	0.0008892
	Intermediate flax	0.0009875
Selection status	Fiber cultivars and breeding lines	0.0003782
	Kryazhs	0.0002771
	Fiber landraces	0.0003034
	Linseed cultivars and breeding lines	0.000638
	Linseed landraces	0.0005433
Geography	Russia	0.000802
	East Europe	0.0008585
	West Europe	0.0009516
	East Asia	0.0008943
	North America	0.0009024

### Selection Signals

**FIGURE 5:**
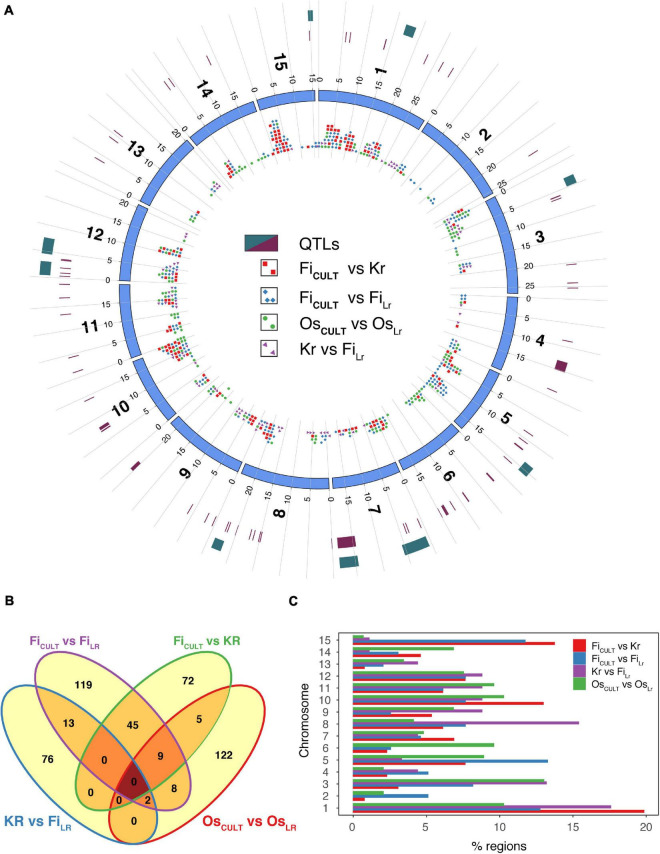
Genomic regions under selection. **(A)** Circos diagram showing genome-wide selective sweep loci identified in different comparisons, with color codes assigned as follows: blue diamonds, fiber flax cultivars (Fi_*CULT*_) vs. fiber flax landraces (Fi_*LR*_); red squares, fiber flax cultivars vs. kryazhs (Kr); violet arrowheads, kryazhs vs. fiber flax landraces; and green dots, oilseed flax cultivars (Os_*CULT*_) vs. oilseed landraces (Os_*LR*_). Two outer rings depict (from inside to outside) known QTLs (22) shown as maroon rectangles, as well as QTLs overlapping with selective sweep regions (dark green rectangles). **(B)** Venn diagram shows the crossover of genomic regions under selection in different comparisons. **(C)** Fraction of candidate selection sweep regions identified in each comparison per chromosome. Color codes corresponding to different comparisons are identical to those used in panel (a) above.

To identify signals of artificial selection, we computed ROD and Fst statistics, as well as examined the regions identified by both methods (Figure 5A and Supplementary Tables 7–10). Thus, to locate candidate regions and genomic intervals, the logical approach would be to find regions that exhibit substantially reduced diversity (a) in fiber flax cultivars, as compared to either fiber flax landraces or kryazhs, (b) in kryazhs, as compared to fiber landraces, and, finally, (c) in oilseed cultivars, as compared to oilseed landraces. By contrasting the aforementioned groups, we identified a total of 564 candidate regions under selection during the most recent breeding events (Table 2). Of these, 82 were detected in several comparisons (Figure 5B). As expected, taking into account the overall large number of fiber flax varieties in the collection, the possibility of overlap was higher either between fiber flax cultivars and landraces or between fiber flax cultivars and kryazhs. We found 196 and 146 regions associated with artificial selection as fiber cultivars were contrasted to landraces and oil cultivars were set against landraces of the same type, respectively (Table 2 and Figure 5B). The specificity of crop improvement signals identified was quite remarkable, as fiber and oil flax had only 19 loci under improvement selection in common.

**TABLE 2:** Number of shared candidate regions between comparisons.

		Fiber flax			Linseed flax
			
		Cultivars vs. landraces	Cultivars vs. kryazhs	Kryazhs vs. landraces	Cultivars vs. landraces
Fiber flax	Cultivars vs. landraces	196	54	15	19
	Cultivars vs. kryazhs	54	131	0	14
	Kryazhs vs. landraces	15	0	91	2
Linseed flax	Cultivars vs. landraces	19	14	2	146

The chromosome-by-chromosome breakdown of selection sweep signals in fiber flax and oil cultivars revealed further astounding differences (Figure 5C and Supplementary Table 11). For instance, in chromosome 15, we identified 34 and 1 improvement signals in fiber and oilseed cultivars, respectively, whereas, in chromosome 6, the trend was reversed with 7 and 14 signals, respectively. Oilseed improvement signals were also depleted in chromosomes 4 and 8 (15.0 and 14.0% of total improvement signals, respectively). Kryazh breeding led to reduction in diversity in 91 candidate regions, only 15 of which were shared with fiber flax cultivars (Table 2). The selection sweep loci in kryazhs were found in all chromosomes, except in chromosomes 2 and 6 (Figures 5A,C and Supplementary Table 9). Chromosome 13 was depleted in improvement signals in all comparisons.

In addition, 11 candidate selection sweep regions on seven chromosomes overlapped with known Quantitative Trait Loci (QTL)s (Figure 5A and Supplementary Table 12), associated with fatty acid content, plant height, and seed mucilage content (You and Cloutier, 2020). The functional repertoire of genes associated with these regions was quite diverse (Supplementary Tables 7–10), encompassing response to stress, protein kinase activity, cell wall biogenesis, protein ubiquitination, fatty acid biogenesis and transport, hormone signaling, embryo and flower development, disease resistance, and finally transcription.

### Geographic Distribution and Gene Flow

Fiber flax kryazhs are a product of long-term breeding efforts carried out in the environmentally most favorable areas of Russian North-West by local peasantry. The most widespread selection practice was to cut off heads from the tallest plants in sheaves, thus aiming for longer and potentially stronger fiber. It is believed that Russian fiber flax landraces and kryazhs are of pure Indo-Afghan origin (i.e., no genetic contribution from European landraces, where ancestry solely lies in the fields of Fertile Crescent, Supplementary Figure 1; Sinskaya, 1959; Zhukovsky, 1971). Moreover, many are of the opinion that all modern fiber flax varieties have Eastern European origin (Helback, 1959). However, apart from these general notions, no further details or genetic evidence were ever provided.

**FIGURE 6:**
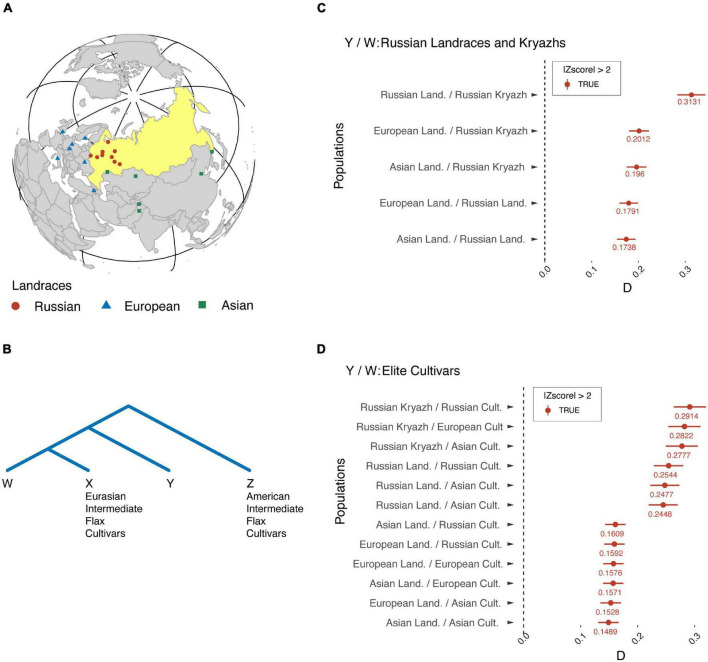
Signatures of gene flow from European and Asian landraces into Russian landraces and kryazhs, as well as from kryazhs and landraces to modern flax fiber cultivars. **(A)** Geographic sites of landrace origin/release. **(B)** Four populations used to calculate *D*-statistics. *Z*, American intermediate flax cultivars; *X*, Eurasian intermediate flax cultivars; *W*, the second sister population; *Y*, the introgressing population. **(C)** *D*-statistics diagram where population *W* corresponds to kryazhs or Russian landraces, while population *Y* stands for landraces of different origin. **(D)** *D*-statistics diagram where population *Y* corresponds to either kryazhs or landraces of different origin. Population *W* represents elite fiber flax cultivars and breeding lines.

To address these questions, we strived to detect signatures of gene flow from landraces that originated in Europe and Asia into Russian landraces and kryazhs, as well as from kryazhs and landraces to modern fiber flax cultivars released in different parts of the world. Consequently, we applied Patterson’s *D*-statistics to measure the relative amount of ancestry of European and Asian landraces in Russian landraces and to test modern fiber flax varieties for admixture from landraces and kryazhs. As indicated in Figure 6, the values of *D*-statistics for each test and comparison were positive, thus indicating that a genomic introgression in kryazhs comes not only from Asian and Russian landraces but also from European landraces. However, it is notable that the contribution of European and Asian landraces to kryazhs was lower, as compared to Russian landraces. Furthermore, the ancestry of modern fiber flax varieties lies in both landraces of different origins, as well as in kryazhs. However, the ancestry contribution of Russian landraces and kryazhs to the modern fiber flax varieties is much higher than the contribution of European and Asian landraces.

**FIGURE 7:**
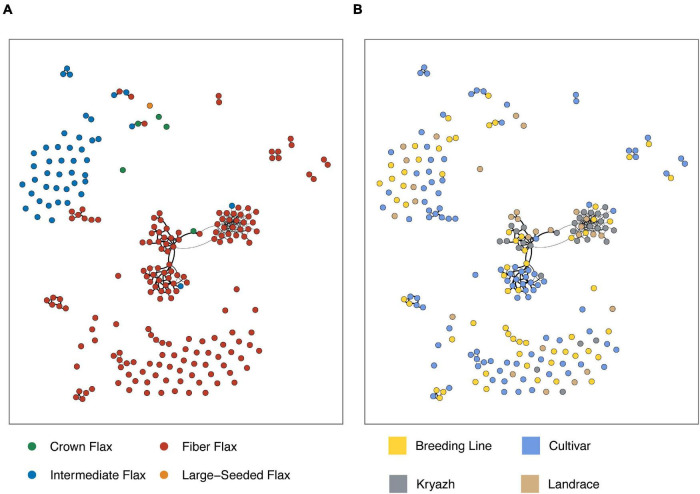
Kinship graph constructed for selected flax samples (pi_hut threshold ≥ 0.5) using IBD. Nodes are flax accessions, while edges represent first-degree relations. Edges thickness reflects pairwise pi_hat values. Edges were further filtered to show those with pi_hat ≥ 0.7547 only (see the “Materials and Methods” section for details). **(A)** Node color corresponds to morphotypes. **(B)** Node color encodes breeding status.

To get further insight into the relationship between kryazhs and modern fiber flax varieties, we constructed first-degree relation network using IBD (Figure 7 and Supplementary Figure 5). Fiber flax accessions were characterized with a higher number of first-degree relations than oilseed accessions. Moreover, kryazhs exhibited a higher degree of connectivity with Russian varieties (42% of first-degree connections), as compared to cultivars from Europe (22%) and Asia (14%).

## Discussion

An extensive characterization of flax genetic diversity is of paramount importance for the long-term sustainability of flax production and diversification, as well as for the overall success of its breeding programs. Recently, a substantial progress has been made in the field, as several national flax genetic studies were published. Thus, flax collections maintained by the Plant Gene Resources of Canada (Diederichsen et al., 2012; Soto-Cerda et al., 2018; You et al., 2018) and the United States National Plant Germplasm System (Guo et al., 2019) and available through the All India Coordinated Research Program of Linseed (Chandrawati et al., 2017) were characterized genetically. Despite good coverage, much of flax diversity remains uncaptured. To fill the gap, we present a genetic characterization of a core collection of flax (306 accessions) maintained by the Russian Federal Research Center for Bast Fiber Crops. Incidentally, this collection, which is one of the finest collections of the world, includes flax varieties from Eurasia with a large proportion of heritage Russian landraces. As a result of the whole genome sequencing effort (mean 10 × coverage), 3,416,829 biallelic SNPs were identified. The genetic variation documented in this study is far more abundant as compared to those reported previously, most likely due to a large number of accessions analyzed and deeper sequencing.

Interestingly, and in contrast to previous studies (Soto-Cerda et al., 2018; Guo et al., 2019; Zhang et al., 2020) that failed to differentiate between oil and fiber flax types, we observed significant population differentiation between oilseed and fiber morphotypes. The application of UMAP algorithm facilitated delineation of the accessions into three groups (Figure 2A and Supplementary Figure 2A), of which the first group consisted of fiber breeding lines and cultivars, the second group was comprised of nearly all kryazhs, and the third group mainly included linseed accessions. The ADMIXTURE analysis yielded four subpopulations in the dataset (Figure 2B). The result generally aligned with the partitioning of the genetic data into three groups obtained with the UMAP algorithm. Of admixed subpopulations, two (red and violet) also formed two distinct groups, as identified by the UMAP algorithm. However, they exhibited weak differentiation suggesting a common breeding history of fiber flax accessions associated with the groups (Figure 2C). Moreover, kinship analysis (Supplementary Figure 4) indicated that fiber flax accessions have strong familial relatedness. It should also be noted that the accessions from UMAP groups 1 and 2 appeared to be partially intermixed on the phylogenetic tree constructed using all 306 accessions (Figure 3A and Supplementary Figure 3), which emphasized their genetic relatedness. Fiber flax and oilseed accessions form two well-separated clades on the tree (Figure 3A). The vast majority of kryazh accessions clustered together within the fiber flax accession clade. Landraces were interspersed between accessions of both clades, reflecting post-domestication diversification of germplasm. Importantly, we did not observe clear clustering of accessions by country of release indicating substantial movement of germplasm. Noteworthy, in one of the earlier studies, Sertse et al. (2019) revealed four well genetically differentiated groups of temperate, South Asian, Mediterranean, and Abyssinian flax. For obvious reasons, as our dataset contained very few accessions from the Mediterranean and South Asia (nine and three genotypes, respectively) and, unfortunately, did not have any accessions from Abyssinia, we were not able to comment on the ecogeographic clustering of genotypes revealed in the analysis by Sertse et al.

In line with the observed significant allelic admixture in the UMAP group composed of mainly oilseed accessions (i.e., group 3) and with results from other studies (Xie et al., 2018), higher variation was observed in oilseed varieties and landraces as compared to fiber flax genotypes. Also, different morphotypes associated with the oilseed group exhibited a surprising genetic homogeneity. However, this result should be interpreted with caution, as both large seeded and crown accessions are far outnumbered by the intermediate morphotype. Importantly, kryazhs appeared to be the least diverse among all genotypes (Figure 4B). Accessions released in Western Europe are the most diverse, followed by other accession groups, namely, North American, East Asian, East European, and, finally, Russian specimens (Table 2). However, the difference in nucleotide diversity between the most contrasting groups was moderate indicating extensive exchange of germplasm between countries and breeding programs.

Various approaches to population structure analysis applied in this study demonstrated the substantial genetic differentiation between oilseed and fiber flax groups. In addition, all fiber flax accessions shared a common genetic background. Although kryazhs were perceived as Russian heritage landraces, this group was genetically distinct and stood out from the rest of the fiber flax landraces. Our results signified the importance of further in-depth integrative efforts aimed to uncover yet unknown fine structure of worldwide flax populations.

Linkage disequilibrium is an important population genetic parameter helping achieve high-accuracy marker-assisted selection and providing insight into the breeding history of the crop. The rate of LD decay is a factor of many variables. This is especially true in the case of selfing organisms, where LD may change dramatically depending on effective population size, due to genetic drift, migration, or selection. We hypothesized that the largest extent of the LD decay observed in kryazh samples is a result of long-lasting mass selection for traits associated with fiber quality and close kinship (Figure 4A). Since the LD decay in sequenced flax accessions is lower as contrasted with cultivated soybean and rice, it is reasonable to expect that the process of screening for casual genes regulating important agronomic traits will be simplified, thus offering incredible opportunities to speed up selection programs for crop breeders.

Further in-depth analyses were conducted to highlight genomic regions affected by recent breeding efforts (Figures 5A,B). An apparent difference in the number of loci under selection in oilseed and fiber flax varieties was observed. For example, in chromosomes 6 and 15, the discrepancy was most pronounced, as they were enriched for oil- and fiber-related improvement signals, respectively. Importantly, the nucleotide diversity between oilseed and fiber groups in these two chromosomes was one of the largest (Supplementary Table 11). Minor overlap between oilseed-specific and fiber-specific selection sweep regions was also detected by Zhang et al. (2020).

In light of the genetic similarity of all fiber flax accessions, it was of interest to compare cultivars with landraces, as well as cultivars with kryazhs. Yet again, it is highly likely that peasantry breeding practices led to the emergence of kryazh varieties and unwittingly resulted in selection for different crop improvement signals than those produced with modern varieties. Indeed, kryazhs shared a small number of crop improvement signals with fiber flax landraces (Figure 5B), which was likely reflected in different requirements for fiber flax improvement in the modern breeding era in light of changing climate and emerging new fields of applications. There was no significant overlap in regions of reduced diversity identified in each of the four contrasts considered, with the noticeable exception of 45 regions shared between fiber flax cultivars and kryazhs and between fiber flax cultivars and fiber landraces, thus suggesting selective breeding for similar traits.

Eleven candidate regions on seven chromosomes overlapped with known QTLs (Figure 5A), associated with fatty acid content, plant height, and seed mucilage content (You and Cloutier, 2020). Interestingly, regions overlapping with fatty acid-associated QTLs were identified not only when contrasting oilseed flax cultivars with oilseed landraces but also when comparing the fiber flax accession groups. This may be indicative of potential pleiotropic effect of SNPs located in the regions. Candidate improvement regions intersect with genes involved in response to stress, protein kinase activity, cell wall biogenesis, protein ubiquitination, fatty acid biogenesis and transport, hormone signaling, embryo and flower development, and disease resistance, which implies selection for oil- and fiber-related traits, as well as against key biotic and abiotic stressors.

In this study, for the first time, we attempted to extensively characterize kryazhs, Russian heritage landraces, to shed light on their breeding history and their relationship with modern fiber flax varieties. Our results provide significant evidence of introgression of genetic material from Asian, Russian, and, importantly, European landraces into kryazhs, supporting the hypothesis of mixed origin of kryazhs from both Indo-Afghan diversity center and Fertile Crescent. Kryazhs breeding history goes hand in hand with the development of modern fiber flax varieties. Although the latter ancestors include landraces of different origins, the contribution from Russian landraces and kryazhs is the most significant (Figures 6C,D). In addition, close ties between kryazhs and modern fiber flax cultivars are evidenced by kinship graph (Figures 7A,B and Supplementary Figure 4). The accessions of kryazhs exhibit a substantial number of first-degree relations with the modern fiber flax cultivars and breeding lines. Nevertheless, as kinship coefficient heatmap presented in Supplementary Figure 4 shows, kryazhs feature strong familiar relatedness that explains both their low genetic diversity and clustering on phylogenetic tree.

## Data Availability Statement

The datasets presented in this study can be found in online repositories. The names of the repository/repositories and accession number(s) can be found below: https://www.ebi.ac.uk/eva/, ERZ2775743.

## Author Contributions

MS: conceptualization. MD, AK, and AS: methodology. AK: software. MB and SS: investigation. TR: resources. MB: data curation. MS, AS, and AK: writing. AK and AS: writing—review and editing. MD and AS: visualization. MS: supervision and funding acquisition. All authors read and approved the final manuscript.

## Conflict of Interest

The authors declare that the research was conducted in the absence of any commercial or financial relationships that could be construed as a potential conflict of interest.

## Publisher’s Note

All claims expressed in this article are solely those of the authors and do not necessarily represent those of their affiliated organizations, or those of the publisher, the editors and the reviewers. Any product that may be evaluated in this article, or claim that may be made by its manufacturer, is not guaranteed or endorsed by the publisher.

## Supplementary Material

Supplementary Figure 1 A map illustrating two hypotheses on the origins of Russian landraces and kryazhs.

Supplementary Figure 2 Grouping of accessions and cluster validation. **(A)** A result of UMAP-assisted K-means clustering of accessions presenting three clusters (i.e., groups). **(B)** Silhouette plot showing the separation distance between the resulting clusters. The average silhouette score across all clusters is plotted as a dashed red line.

Supplementary Figure 3 A maximal likelihood phylogenetic tree constructed from genome-wide SNPs. The outermost ring depicts distribution of accession in accordance with UMAP-derived groups.

Supplementary Figure 4 A heatmap representation of kinship coefficients calculated between accession pairs. The coefficient values are shown in the shades of blue. Side panels show breeding status and morphotype for each accession.

Supplementary Figure 5 Kinship graphs constructed for flax accessions broken down by selection status and colored by morphotype. Nodes are accessions, while edges represent first-degree relations (pi_hat > 0.5). Edge thickness reflects pairwise pi_hat values. **(A)** Kryazhs, **(B)** landraces, and **(C)** breeding lines and cultivars.
