# Supplementary figures and images for: The Genetic Landscape of Fiber Flax
Source: Front Plant Sci. 2021 Dec 7;12:764612. doi: 10.3389/fpls.2021.764612 (PMC8691122; doi:10.3389/fpls.2021.764612)

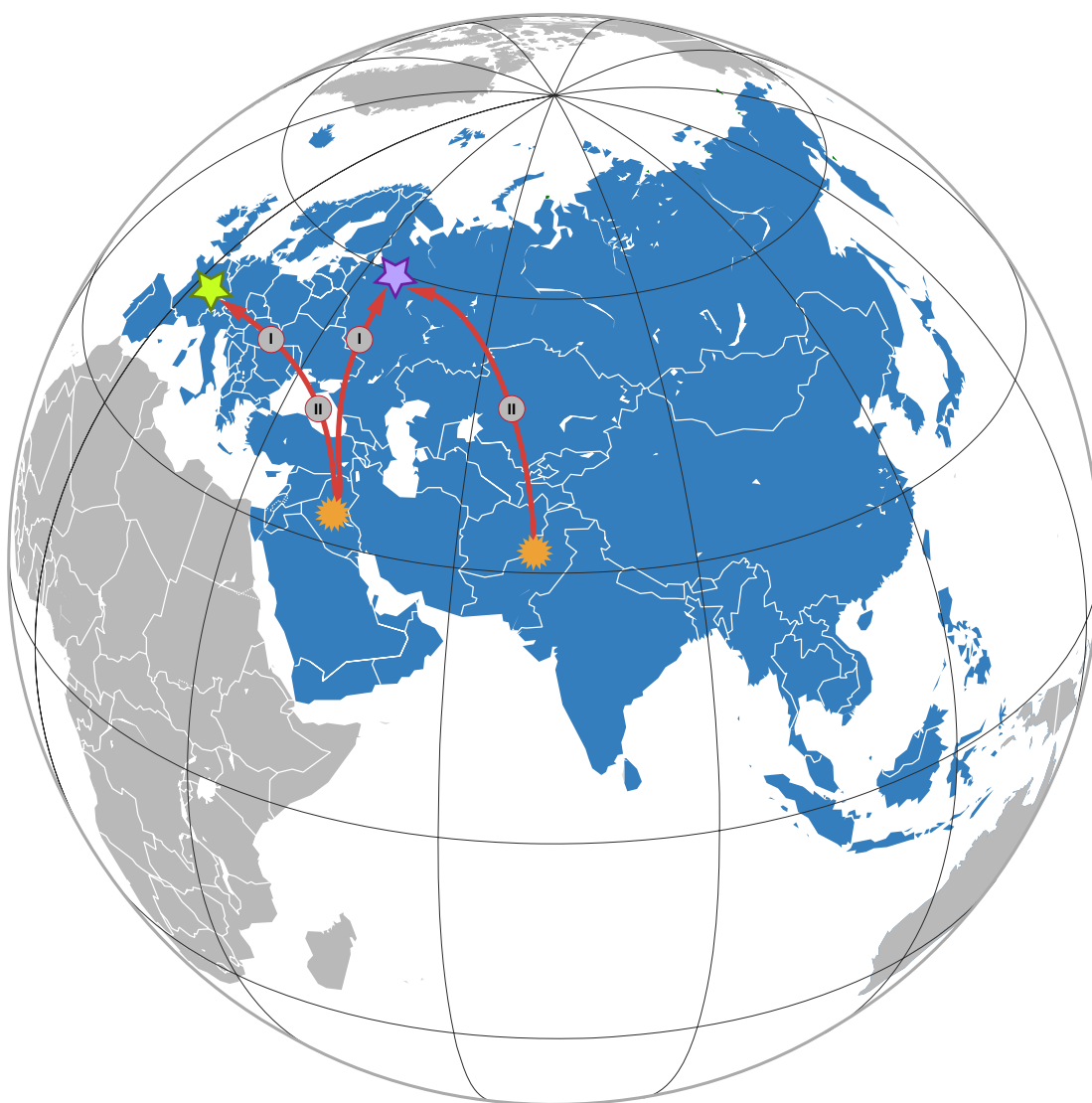

- 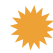 Flax Origin
- 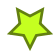 European Varieties
- 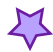 Russian Landraces & Kryazhs

**Supplemental Figure 1**

Supplement: Supplementary Figure 1 — A map illustrating two hypotheses on the origins of Russian landraces and kryazhs. [file Data_Sheet_1.PDF]

**a**

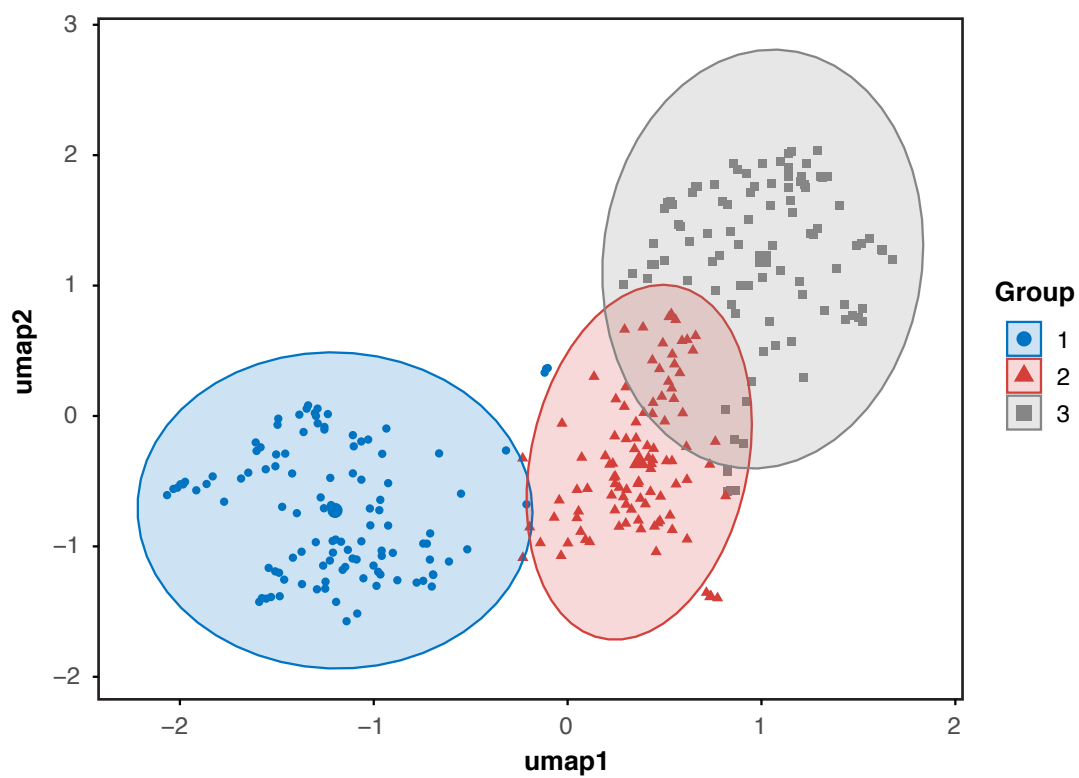

**b**

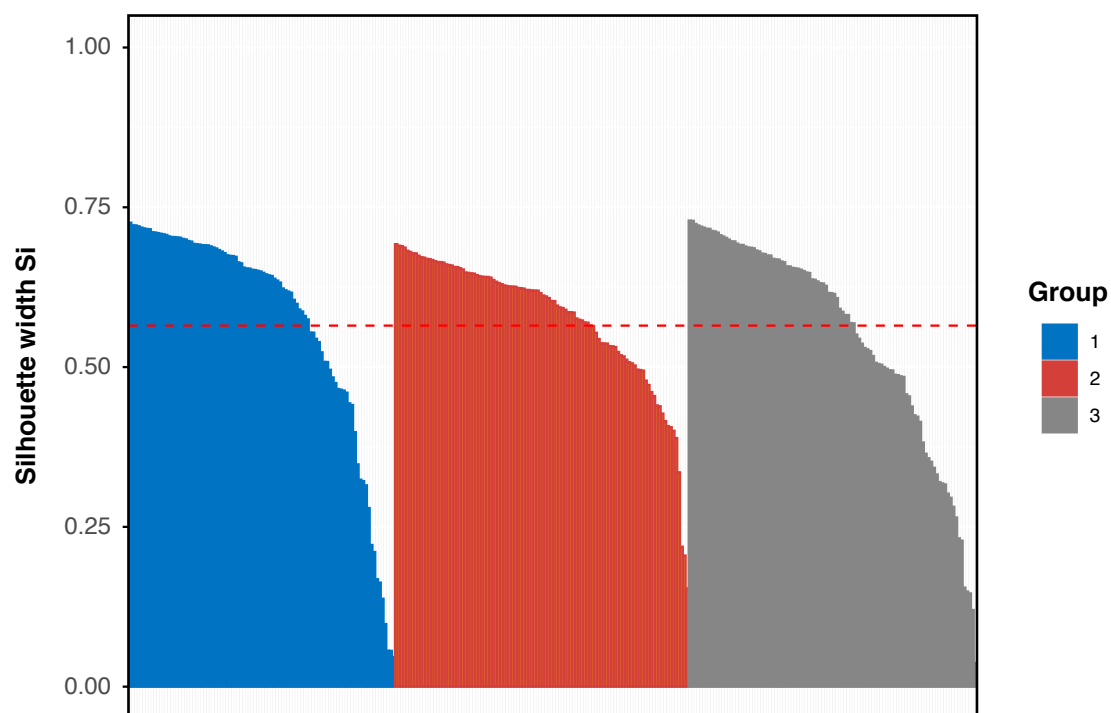

**Supplemental Figure 2**

Supplement: Supplementary Figure 2 — Grouping of accessions and cluster validation. (A) A result of UMAP-assisted K-means clustering of accessions presenting three clusters (i.e., groups). (B) Silhouette plot showing the separation distance between the resulting clusters. The average silhouette score across all clusters is plotted as a dashed red line. [file Data_Sheet_2.PDF]

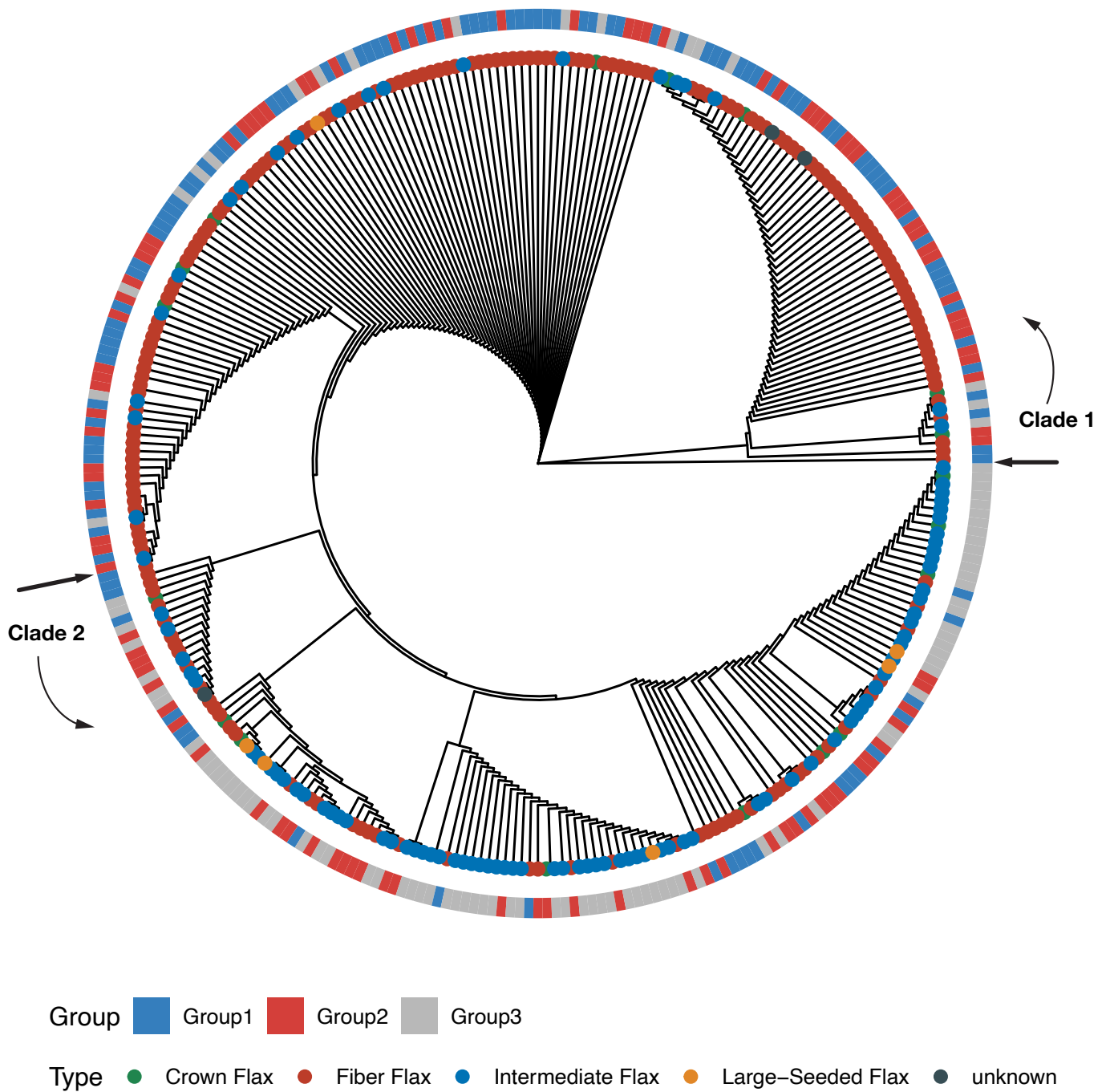

Supplemental Figure 3

Supplement: Supplementary Figure 3 — A maximal likelihood phylogenetic tree constructed from genome-wide SNPs. The outermost ring depicts distribution of accession in accordance with UMAP-derived groups. [file Data_Sheet_3.PDF]

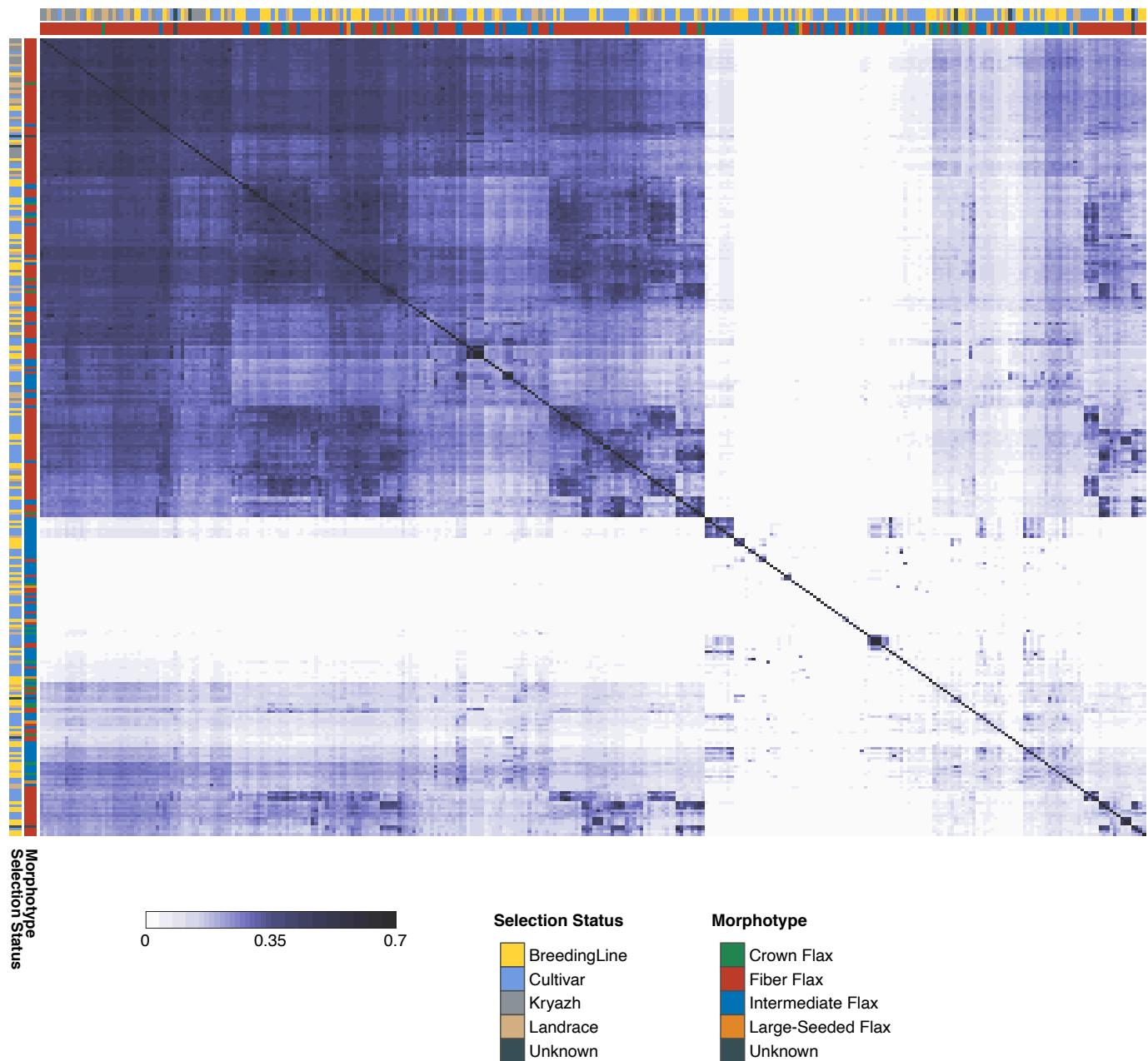

Supplemental Figure 4

Supplement: Supplementary Figure 4 — A heatmap representation of kinship coefficients calculated between accession pairs. The coefficient values are shown in the shades of blue. Side panels show breeding status and morphotype for each accession. [file Data_Sheet_4.PDF]
