# Supplementary material for: The Genetic Landscape of Fiber Flax
Source: Front Plant Sci. 2021 Dec 7;12:764612. doi: 10.3389/fpls.2021.764612 (PMC8691122; doi:10.3389/fpls.2021.764612)
Supplement: Supplementary Figure 5 — Kinship graphs constructed for flax accessions broken down by selection status and colored by morphotype. Nodes are accessions, while edges represent first-degree relations (pi_hat > 0.5). Edge thickness reflects pairwise pi_hat values. (A) Kryazhs, (B) landraces, and (C) breeding lines and cultivars. [file Data_Sheet_5.PDF]

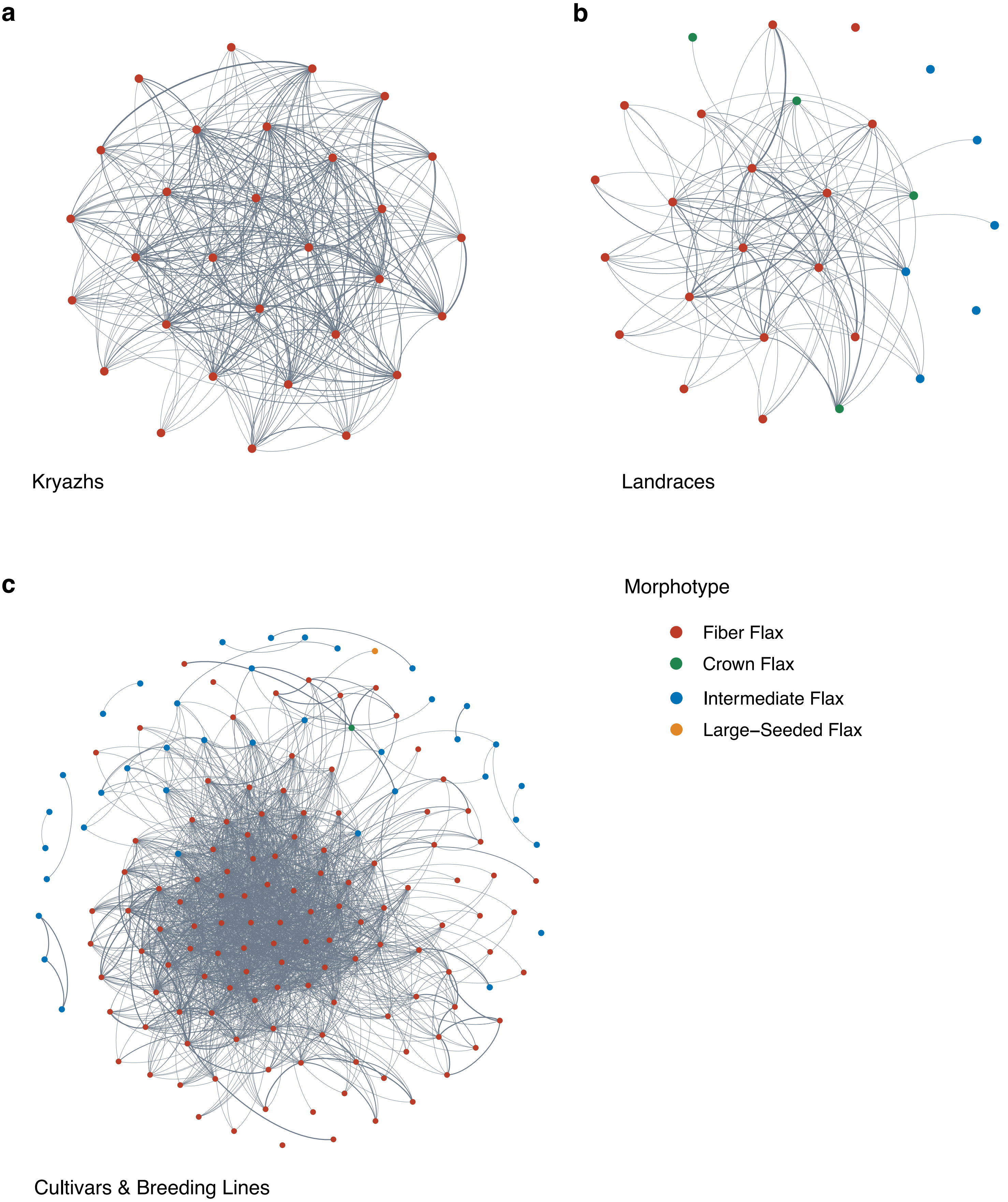

Supplemental Figure 5
